# Measurable residual disease in childhood B-cell acute lymphoblastic leukemia

**DOI:** 10.1097/BS9.0000000000000112

**Published:** 2022-10-10

**Authors:** Robert Peter Gale

**Affiliations:** 1 Haematology Research Centre, Department of Immunology and Inflammation, Imperial College, London W12 ONN, UK

In a recent report in *Nature Cell Biology* (2022. 24:242–52) Zhang and coworkers discuss comparative genetic and biochemical features of leukemia cells from children with B-cell acute lymphoblastic leukemia (ALL) obtained at diagnosis, in remission, and at relapse.^1^ The authors analyzed large numbers of single-cell transcriptomes looking for dynamic changes and simultaneously, B-cell receptor sequences. They report that in contrast to leukemia cells at diagnosis, those at relapse shifted to a poorly-differentiated state. Changes in residual leukemia cells in remission were more complicated. Differential functional analyses highlighted activation of the hypoxia pathway in residual leukemia cells which correlated with drug resistance which was reversible with appropriate drug interventions in in vitro and in vivo models. The authors suggest this might be a therapy approach to eradicating measurable residual disease (MRD) in childhood B-cell ALL.

This is a data dense article which requires understanding a machine learning algorithm. I suggest putting aside at least 5 hours to read and understand the text and supplement. I had to read it twice. This is not something to breeze through while texting on WeChat if you really want to understand the authors’ message and to critique it appropriately.

First, a word on nomenclature. The authors use the term *minimal residual disease*. As John Goldman and I discussed several years ago the correct term is *measurable residual disease*.^2^*Minimal* is a subjective term; *minimal* to 1 person is not necessarily *minimal* to another. What we are considering is what can and cannot be measured in someone in complete histological remission. (Another source of confusion; remissions are *histological*, not *morphological*. Morphology comes from the Greek *μ&z.omicr;ρϕ* which means form, structure or shape, not appearance). As an aside Morpheus was the Greek God of sleep and is the root of the drug name morphine. Lest you think I’m getting lost in semantics please recall the comment from George Orwell: *Slovenliness of our language makes it easier for us to have foolish thoughts.*^3^

For MRD to be meaningful we need to know what technique is used to detect residual leukemia cell, its sensitivity and specificity, accuracy and precision, false-positive and -negative rate, and other parameters. We discuss these issues in considerable detail in two recent articles.^3–6^ The key point in the context of the article by Zhang and coworkers is our MRD tests do not distinguish between residual leukemia cells able or not to cause relapse. For example, in chronic myeloid leukemia (CML) tyrosine kinase-inhibitor-therapy does not eradicate the CML stem cell but some people with CML achieving a sustained deep molecular response can stop therapy without leukemia recurrence, a situation referred to as operational cure.^7,8^

Back to the article by Zhang and coworkers. My purpose is not to discuss the elegant experiments they did which would take considerable space and is best done by studying their article in detail. Rather, my purpose is to try to put their interesting and important findings in context. To know if becoming hypoxic is really how residual leukemia cells escape eradication (or if chemotherapy selects for emergence of hypoxic cells) we would need a prospective study showing a correlation. This article does not provide such data but perhaps the authors have this as their next task. This would be a difficult study to do since we fortunately cure most children with B-cell ALL. Another difficult challenge would be to prove anti-hypoxemic drugs improve therapy outcomes.

Another interesting question is how we cure children with B-cell ALL. Most children achieving a histological remission with conventional therapy with corticosteroids, vincristine, cytarabine, doxorubicin, L-asparaginase, and etoposide then receive 2 to 3 years of so-called *maintenance* therapy with 6-mercaptopurine and methotrexate. But what exactly does maintenance therapy do? It is most unlikely these drugs typically given at low doses kill ALL *stem cells.* More likely, B-cells, normal and leukemia, are fated to die and maintenance therapy simply delays leukemia relapse sufficiently so these cells commit suicide.^9^

I was especially interested in the authors’ use of a machine learning algorithm to classify stages of B-cell development (described in METHODS). For those unfamiliar with this technique this is a supervised approach where there are learning and validation cohorts. Reproducibility was high but correct attribution accuracy always depends on accuracy of labeling of the training dataset.

In summary, I recommend a careful reading of this important study. Much of the methods will be beyond the comfort zone of most hematologists. As such, struggling through and trying to understand the techniques may be as or more important than the conclusion. If you are going to print the article out (a good idea for reading on the high speed train) be sure to use a color printer or you will be lost. Finally, this article is a striking example of the increasingly high quality of biomedical research in China. I congratulate the authors. I wish they had submitted it to *Leukemia.*

## ACKNOWLEDGMENT

RPG acknowledges support from the National Institute of Health Research (NIHR) Biomedical Research Centre funding scheme.
